# Cardiogenic shock in pregnancy: Prolonged axillary Impella 5.5 support during pregnancy and postpartum as bridge to heart transplantation

**DOI:** 10.1016/j.jhlto.2026.100620

**Published:** 2026-06-30

**Authors:** Shahar Laks, Rebecca Pierce-Williams, Ngina Connors, Kelecia Brown, Jennifer Philips, Diane Holmes, Catarina Canha, Nicole Cyrille-Superville, Esther Kim, Monique Oye

**Affiliations:** a Department of Cardiovascular Medicine, Atrium Health Sanger Heart & Vascular Institute, Charlotte, North Carolina; b Department of Maternal-Fetal Medicine, Atrium Health, Charlotte, North Carolina

**Keywords:** mechanical circulatory support, advanced heart failure, cardio-obstetrics, pregnancy, transplant, permpartum cardiomyopathy, impella

## Abstract

**Background:**

Cardiogenic shock during pregnancy carries substantial maternal and fetal morbidity and mortality, and guidance on temporary mechanical circulatory support (tMCS) in this setting remains limited.

**Case summary:**

A 36-year-old woman at 21 weeks' gestation presented with severe dilated cardiomyopathy (ejection fraction 20%, LVIDd 8.0 cm), cardiogenic shock (cardiac index 1.7 L/min/m²), and recurrent ventricular arrhythmias. She declined termination of pregnancy. An axillary Impella 5.5 was placed for hemodynamic stabilization with rapid improvement in cardiac output and filling pressures. Progressive ventricular arrhythmias at 23 weeks and 3 days necessitated urgent cesarean delivery; the neonate did not survive. The patient was supported with the Impella 5.5 for 66 days and underwent successful orthotopic heart transplantation.

**Discussion:**

To our knowledge, this is one of the longest reported durations of Impella 5.5 support initiated during pregnancy and continued into the postpartum period. This case highlights important considerations that may inform individualized multidisciplinary decision-making on temporary mechanical circulatory support selection in pregnancy-associated cardiogenic shock.

## History of presentation

A 36-year-old G11P6046 woman at 21 weeks' gestation presented to the hospital with progressive dyspnea over several weeks. Initial laboratory evaluation demonstrated elevated B-Type Natriuretic Peptide (BNP) of 303 pg/mL. Transthoracic echocardiogram revealed a left ventricular ejection fraction (LVEF) of 20%, with marked left ventricular remodeling, including a left ventricular internal diameter in diastole of 8.0 cm, suggesting a chronic disease process despite the recent onset of overt symptoms. Severe secondary functional mitral regurgitation was also noted. Right ventricular function was moderately reduced on echocardiogram.

Right heart catheterization revealed elevated filling pressures (RA 14 mmHg, PCWP 30 mmHg), a cardiac index of 1.7 L/min/m², and pulmonary artery pressures of 44/32 mmHg (mean 38 mmHg) with pulmonary vascular resistance (∼2.7 WU).

Continuous telemetry monitoring revealed recurrent episodes of sustained ventricular tachycardia. Fetal ultrasound was performed and showed normal fetal anatomy and appropriate fetal size.

## Differential diagnosis

At presentation, chronic left ventricular dilation (LVIDd 8.0 cm) and early gestational age at presentation suggested dilated non-ischemic cardiomyopathy, potentially representing an undiagnosed episode of peripartum cardiomyopathy from her last pregnancy. She had not undergone cardiac evaluation before the current pregnancy. Genetic dilated cardiomyopathy was considered as a possible etiology. She had a negative family history and genetic testing performed with Invitae comprehensive cardiomyopathy panel during admission was negative, although a negative panel does not exclude a genetic contribution. Myocarditis, ischemic cardiomyopathy, and toxin-mediated cardiomyopathy were considered less likely due to the absence of viral prodrome, chest pain, ischemic symptoms, or substance use.

## Management

The patient was managed by a multidisciplinary cardio-obstetrics team including advanced heart failure cardiologists, maternal-fetal medicine, cardiac anesthesia, cardiothoracic surgery, neonatology, and pediatric palliative care. She was counseled on the significant fetal and maternal risks associated with continuation of the pregnancy in the setting of advanced cardiomyopathy, including risk of maternal death, fetal death, or both. The risks of delivery at peri-viability were discussed, including neonatal risks and risk of neonatal demise. She declined termination of pregnancy. She requested pregnancy continuation as long as possible and all possible neonatal interventions at delivery. Based on the patient’s wishes, she was monitored closely with weekly multidisciplinary meetings for care coordination. Her clinical course was complicated by worsening hypotension, and recurrent sustained ventricular arrhythmias requiring synchronized cardioversion and anti-arrhythmic therapy with IV lidocaine infusion. Guideline-directed medical therapy was limited by hypotension, and ventricular arrhythmias further limited the use of inotropes. Given the severity of her hemodynamic compromise consistent with SCAI Stage D cardiogenic shock, mechanical circulatory support (MCS) was pursued with an axillary Impella 5.5 device to provide temporary left ventricular support.

The patient underwent placement of an axillary Impella 5.5 via right axillary artery cutdown. Following initiation of Impella 5.5 support, there was a rapid improvement in hemodynamics. Cardiac output increased to 5.3 L/min with a cardiac index of 2.7 L/min/m². Filling pressures improved, with a right atrial pressure of 4 mmHg, pulmonary artery pressure of 24/12 mmHg, pulmonary capillary wedge pressure of 11 mmHg.

Vasoactive and inotropic support were not required following device initiation. Serum lactate, which was mildly elevated at 2.9 mmol/L prior to support, normalized to 0.9 mmol/L.

During prolonged Impella 5.5 support, anticoagulation was maintained with systemic unfractionated heparin targeting an activated partial thromboplastin time (aPTT) of 60 to 80 s. A bicarbonate-based purge solution was utilized.

The device was maintained at performance level P8 with flows of approximately 4.8 L/min. Device position and function were assessed with serial echocardiography and continuous hemodynamic monitoring.

The patient underwent regular surveillance for hemolysis and device-related complications. Surveillance labs included plasma-free hemoglobin, lactate dehydrogenase, haptoglobin, and total bilirubin. The axillary approach allowed mobilization and participation in physical therapy during the support period. No device malfunction, pump thrombosis, hemolysis, stroke, vascular injury, or need for device exchange occurred during support.

Continuous fetal monitoring was planned to begin at 24 weeks 0 days, with efforts to extend gestation to at least 28 weeks if patient remained stable on Impella support. She was given betamethasone at 22 weeks and 5 days given desire for neonatal intervention at delivery and risk of needing emergent preterm delivery.

At 23 weeks and 3 days on hospital day 12, the patient had an episode of ventricular fibrillation with successful direct current cardioversion. Given progression to unstable ventricular arrhythmias, the decision was made to proceed with urgent cesarean delivery with cardiac anesthesia support. The patient underwent an uncomplicated primary low transverse cesarean section under general anesthesia. Her postoperative course was complicated by multiple abdominal wall hematomas, confirmed on CT abdomen.

## Outcome and follow-up

**Figure 1:**
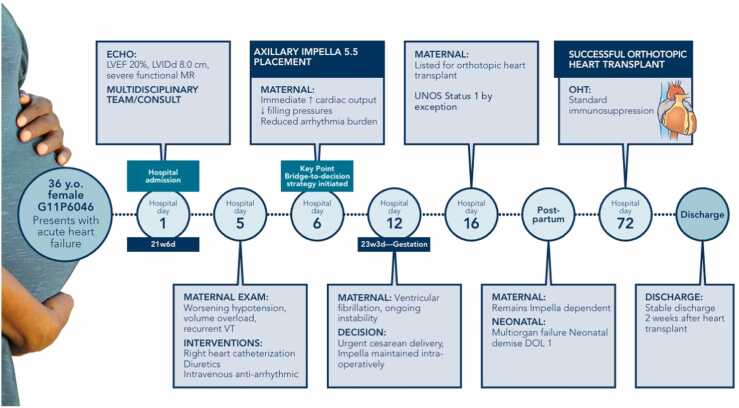
Clinical timeline of cardiogenic shock in pregnancy and postpartum managed with axillary Impella 5.5

Postpartum, the patient remained dependent on mechanical circulatory support and was evaluated for advanced heart failure therapies. Comprehensive transplant assessment demonstrated no absolute contraindications to transplantation. Calculated panel reactive antibody (cPRA) was 74%, and donor crossmatch testing was negative. Following delivery, she was listed at high-priority United Network for Organ Sharing Status 1 by exception on hospital day 16, supported by ongoing hemodynamically significant arrhythmias despite medical therapy and dependence on mechanical circulatory support. She underwent successful orthotopic heart transplantation 56 days later (Figure 1).

The patient remained hospitalized following delivery, supported with the axillary Impella 5.5 while awaiting transplantation. The patient remained supported with axillary Impella 5.5 for a total of 66 days. On hospital day 72, she underwent successful orthotopic heart transplantation without major complications. Pathology was consistent with dilated cardiomyopathy, characterized by severe biventricular dilation, myocyte stretch attenuation, and focal mild disarray. Unfortunately, the neonate’s course was complicated by multiorgan failure and the neonate passed on day of life 1. Neonatal birth weight was 580 g with Apgar scores of 2 and 3 at 1 and 5 min, respectively. The psychological burden of prolonged critical illness and neonatal demise contributed to grief, anxiety, and depression. Supportive care was provided by the primary teams with added support from psychiatry consultation.

## Discussion

Cardiogenic shock during pregnancy is rare but carries substantial maternal and fetal morbidity and mortality.^1^ To the best of available evidence, this is among the first reported cases of prolonged axillary Impella 5.5 support during pregnancy and postpartum as a bridge to heart transplantation.

This case highlights important considerations that may inform individualized multidisciplinary decision-making regarding temporary mechanical circulatory support selection in pregnancy-associated cardiogenic shock. As a single case report, these observations should be viewed as hypothesis-generating rather than prescriptive guidance. Key considerations include (1) gestational age and fetal viability, (2) likelihood of myocardial recovery, (3) severity of hemodynamic compromise and arrhythmia burden, (4) anticipated duration of support, and (5) physiologic constraints unique to pregnancy. In patients presenting in early gestation with severe cardiomyopathy and low likelihood of recovery, strategies that prioritize hemodynamic stabilization and allow for prolonged support and evaluation for advanced therapies may be favored over short-term rescue approaches aimed at myocardial recovery.

In our patient, the use of axillary Impella 5.5 support allowed for maternal stabilization in severe cardiogenic shock in pregnancy. However, despite efforts to prolong gestation, neonatal outcomes were poor, emphasizing the significant challenges in balancing maternal and fetal risks in early gestation.

Current guidelines and scientific statements emphasize early recognition and multidisciplinary management of cardiovascular disease in pregnancy but offer limited guidance regarding the use of temporary mechanical circulatory support (tMCS) due to limited data.^2^ Temporary support modalities such as intra-aortic balloon pump (IABP) and venoarterial extracorporeal membrane oxygenation (VA-ECMO) have been described in pregnancy; however, prolonged support strategies remain poorly defined.^2^ The Impella 5.5 is a temporary ventricular support device capable of providing full left ventricular unloading and cardiac output support.^3^ Its axillary placement allows for patient mobilization, which is particularly advantageous in scenarios requiring prolonged stabilization.

The 66-day duration of axillary Impella 5.5 support in this case exceeds the FDA-approved duration of 14 days. In the Cardiogenic Shock Working Group (CSWG) multicenter registry, the median duration of Impella 5.0/5.5 support was 12.9 days, with a median of 23.9 days among patients supported beyond 14 days^3^; prolonged support was not associated with linear increase in serious adverse event rates.^4^ Although no device malfunction, pump thrombosis, hemolysis, stroke, vascular injury, or need for device exchange occurred during support, the patient's postoperative course was complicated by abdominal wall hematomas while receiving systemic anticoagulation. Our patient's 66-day support duration demonstrates the feasibility of prolonged support in a highly selected patient.

Recent case series have demonstrated successful use of temporary mechanical circulatory support with IABP and VA-ECMO in peripartum patients as a bridge to postpartum recovery,^5^ particularly at later gestational ages where neonatal outcomes are more favorable. These strategies were carefully considered for our patient; however, the severity of left ventricular dysfunction and electrical instability made meaningful stabilization with IABP alone unlikely.^6^ Venoarterial extracorporeal membrane oxygenation (VA-ECMO) may provide more rapid cardiopulmonary stabilization and has been reported as a bridge to fetal viability in selected peripartum patients.^7^ However, in this patient, VA-ECMO posed several limitations, including increased left ventricular afterload with risk of ongoing ventricular distension, the need for additional left ventricular unloading strategies, susceptibility to aortocaval compression from the gravid uterus, and restrictions on patient mobility and positioning over a potentially prolonged duration of support. In contrast, the axillary Impella 5.5 provided effective ventricular unloading with preserved mobility. While earlier initiation of alternative MCS strategies or escalation to VA-ECMO may theoretically have permitted modest prolongation of gestation, it is uncertain that such approaches would have meaningfully altered neonatal outcomes given the early gestational age at presentation,^8^ severity of maternal illness, and low likelihood of myocardial recovery.

The decision to pursue prolonged temporary mechanical circulatory support as a bridge to transplantation, rather than transition to durable LVAD therapy, was informed by multidisciplinary evaluation and patient-specific factors. Durable LVAD implantation was considered less suitable given recent cesarean delivery complicated by abdominal hematomas and right ventricular dysfunction at presentation with concern for post-LVAD right heart failure. Although right ventricular function was moderately reduced at presentation, RV performance improved following left ventricular unloading and optimization of filling pressures and did not require dedicated right-sided mechanical circulatory support during the hospitalization**.** Given preserved end-organ function and acceptable pulmonary hemodynamics, a strategy of bridge to transplantation with prolonged Impella support was favored.

Maternal-fetal decision-making in this case required continuous multidisciplinary reassessment. Delivery decisions were driven by maternal instability, specifically progressive ventricular arrhythmias and risk of hemodynamic collapse.^9^ This case underscores the ethical and clinical principle that maternal survival remains paramount. It also demonstrates the feasibility of prolonged axillary Impella 5.5 support initiated during pregnancy and continued postpartum in a patient with severe pregnancy-associated cardiogenic shock. While conclusions regarding optimal support strategies cannot be drawn from a single case, this experience highlights factors that may inform multidisciplinary decision-making when prolonged stabilization and evaluation for advanced therapies are needed.

Further investigations are needed to better define optimal management strategies and to inform maternal-fetal decision-making in cases of cardiogenic shock during pregnancy.

## Disclosures

The authors have no relationships with industry to disclose.

## Informed consent

Written informed consent for publication of this case report was provided by the patient.

## Funding

The authors report no external funding for this work.

## Declaration of competing interest

The authors declare that they have no known competing financial interests or personal relationships that could have appeared to influence the work reported in this paper.
